# The effect of active hand movement on visually perceived depth direction

**DOI:** 10.1371/journal.pone.0245000

**Published:** 2021-01-06

**Authors:** Hiroyuki Umemura

**Affiliations:** Human Augmentation Research Center, National Institute of Advanced Industrial Science and Technology (AIST), Kashiwa, Chiba, Japan; Johns Hopkins University, UNITED STATES

## Abstract

This study investigated the effect of active hand movement on the perception of 3-D depth change. In Experiment 1, the 3-D height of an object synchronously changed with the participant’s hand movement, but the 3-D height of the object was incongruent with the distance moved by the hand. The results showed no effect of active hand movement on perceived depth. This was inconsistent with the results of a previous study conducted in a similar setting with passive hand movement. It was speculated that this contradiction appeared because the conflict between the distance moved by the hand and visual depth changes were more easily detected in the active movement situation. Therefore, it was assumed that in a condition where this conflict was hard to detect, active hand movement might affect visual depth perception. To examine this hypothesis, Experiment 2 examined whether information from hand movement would resolve the ambiguity in the depth direction of a shaded visual shape. In this experiment, the distance moved by the hand could (logically) accord with either of two depth directions (concave or convex). Moreover, the discrepancy in the distances between visual and haptic perception could be ambiguous because shading cues are unreliable in estimating absolute depth. The results showed that perceived depth directions were affected by the direction of active hand movement, thus supporting the hypothesis. Based on these results, simulations based on a causal inference model were performed, and it was found that these simulations could replicate the qualitative aspects of the experimental results.

## Introduction

The human brain integrates information from various sensory sources relevant to the perception of a unified event or object. A great deal of research has been dedicated to revealing how the brain integrates information from multiple senses or multiple cues from the same sensory modality. These studies have shown that information from different senses is combined in a statistically optimal fashion that yields a more precise estimation than that derived from either sense alone. For example, Ernst and Banks [1] asked participants to estimate the width of their experimental stimuli based on haptic and visual information and manipulated the reliability of visual information through added noise. Their results showed that haptic information became dominant and the weighting of the visual information decreased since the reliability of the visual information was reduced due to noise. This means that the weighting of each modality changes with the reliability of the signals to obtain an optimal estimate. Such a change in weights can also be observed in more natural settings. Gepshtein and Banks [2] asked observers to estimate the distance between two parallel surfaces based on visual and haptic information from various angles and showed that weights for each type of information were reassigned with the visibility of the intersurface gap.

Another question regarding the integration of information from multiple senses concerns the manner in which the brain determines whether information from different senses should be combined to increase perceptual precision or whether it should not be combined to avoid merging information derived from different objects or events. Gepshtein et al. [3] have shown that spatial coincidence affects the integration of information from visual and haptic signals. They found that discrimination precision was essentially optimal when the signals came from the same location, whereas this discrimination was less precise when the signals originated from different locations. Helbig and Ernst [4] showed that even if the positions of two signals from different senses were spatially separated, the brain combined such signals when prior knowledge of a coincidence was presented. Furthermore, other studies have shown that temporal separation is also a factor that affects the combination of signals [5,6]. For instance, when signals arising from different modalities were presented asynchronously, the information from the respective modalities did not affect the other modality. To some extent, however, the brain can adapt or recalibrate temporal differences between two modalities [7,8]. The discrepancy between estimations from each sense is also used as a criterion to determine whether these estimations will be combined. That is, if two estimations greatly differ, then the brain separates them under the assumption that they do not originate from the same source [9].

Both of these processes, namely, the reweighting process in multiple signal integration and the decision-making process about whether or not signals come from the same source, have been formulated within a Bayesian framework [10–14]. In particular, the latter process has been formulated as the “causal inference model.” This model showed good estimates for experimental results on multimodal integration, such as the ventriloquism effect [12–14].

As described above, many studies have revealed the nature of multimodal integration from various aspects. However, very few studies have investigated situations in which an observer actively deforms the shape of an external object. In everyday situations, we frequently distort or manipulate the shape of an object using either our hands or tools. Integration of visual and haptic information in 3-D perception is involved in such a situation, such as when one kneads clay or cuts an object with a knife. Umemura [15] showed that passive hand movement, synchronized with visual depth movement, modified the perceived direction of visual depth movement under a simulated situation in which the grabbed stylus was automatically moved with a deformed surface as if the stylus was attached to the surface. Even if the moved trajectory was the same as passive movement, there should be several differences between active and passive movement conditions. For example, it is known that active hand movement could produce more precise proprioceptive information [16], there have been few studies about how the precision differentiates the effect of hand movement in the active and passive case. In Experiment 1, it was investigated whether the effect of active hand movement was different from that of passive hand movement in a similar experimental setting as the abovementioned study [15].

## Experiment 1

### Material and methods

#### Apparatus and stimuli

**Fig 1:**
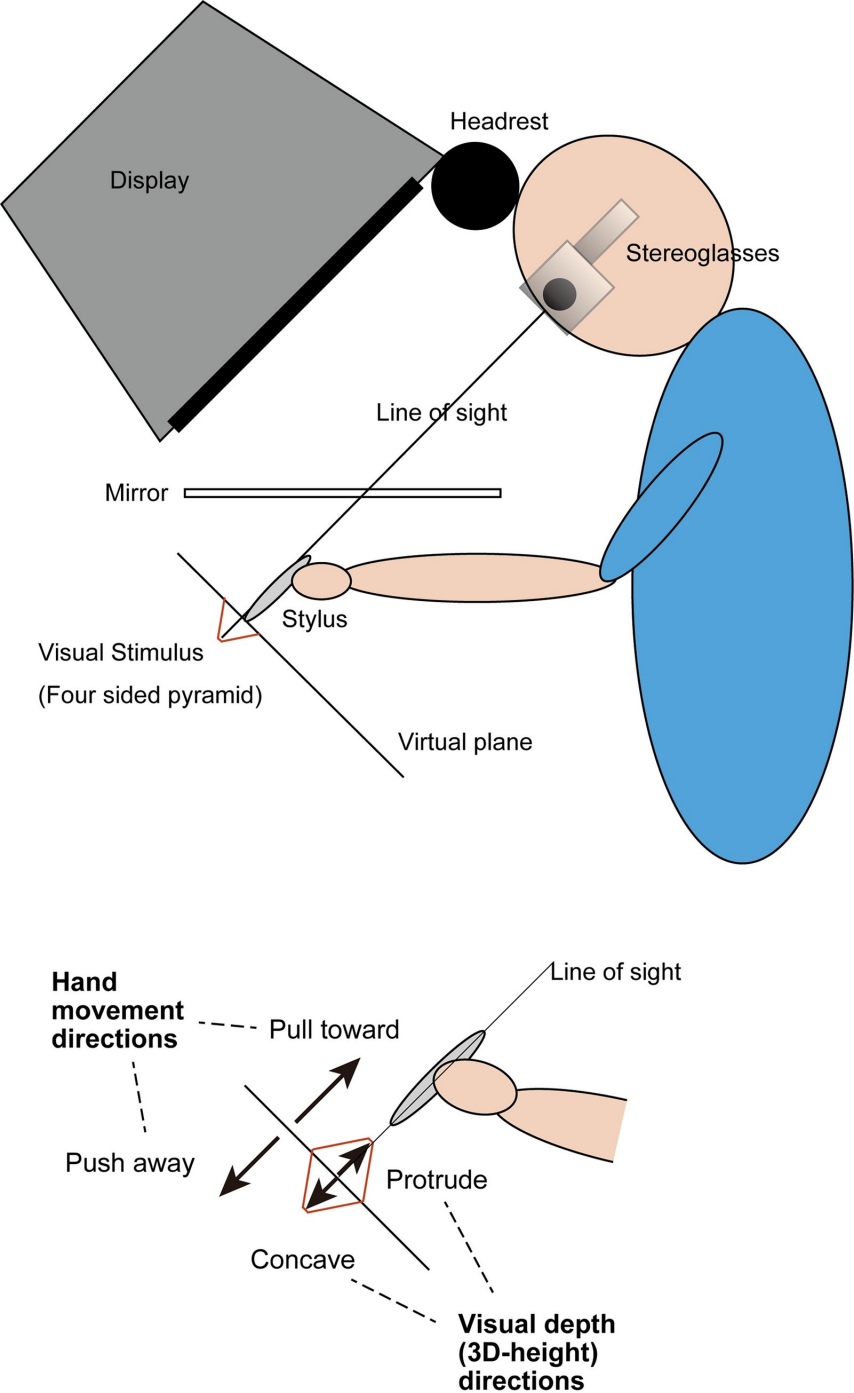
Illustration of experimental setup. Participants binocularly viewed a stimulus in a mirror that reflected the CRT display situated above the mirror, which enabled the cursor positioned in a simulated 3-D space to coincide with the point of the stylus on a haptic device located under the mirror.

In this experiment, a haptic device (PHANToM Desktop, Sensable Technologies Inc.) was used to track the participant’s hand movement and to generate haptic feedback. To coincide a visual space with the haptic workspace, participants binocularly viewed a visual stimulus in a mirror that reflected a cathode ray tube (CRT) display (Viewsonic E90fb, 19 inch, 1024 × 768, 120 Hz refresh rates, 60 Hz for each eye) situated above the mirror. The coordination of the experimental setting is shown in Fig 1. The position of the stylus point of the haptic device was indicated by a cursor in the shape of a blue cone. The height of the cone was 1 cm, and the radius of its base circle was 0.5 cm in the simulated 3-D space. The participant’s hand was beneath the mirror and could not be observed by the participant. The distance between the participant’s eye and the display through the reflection in the mirror was 70 cm. CrystalEyes (StereoGraphics Inc.) liquid crystal shutter glasses were used to present binocular disparity.

**Fig 2:**
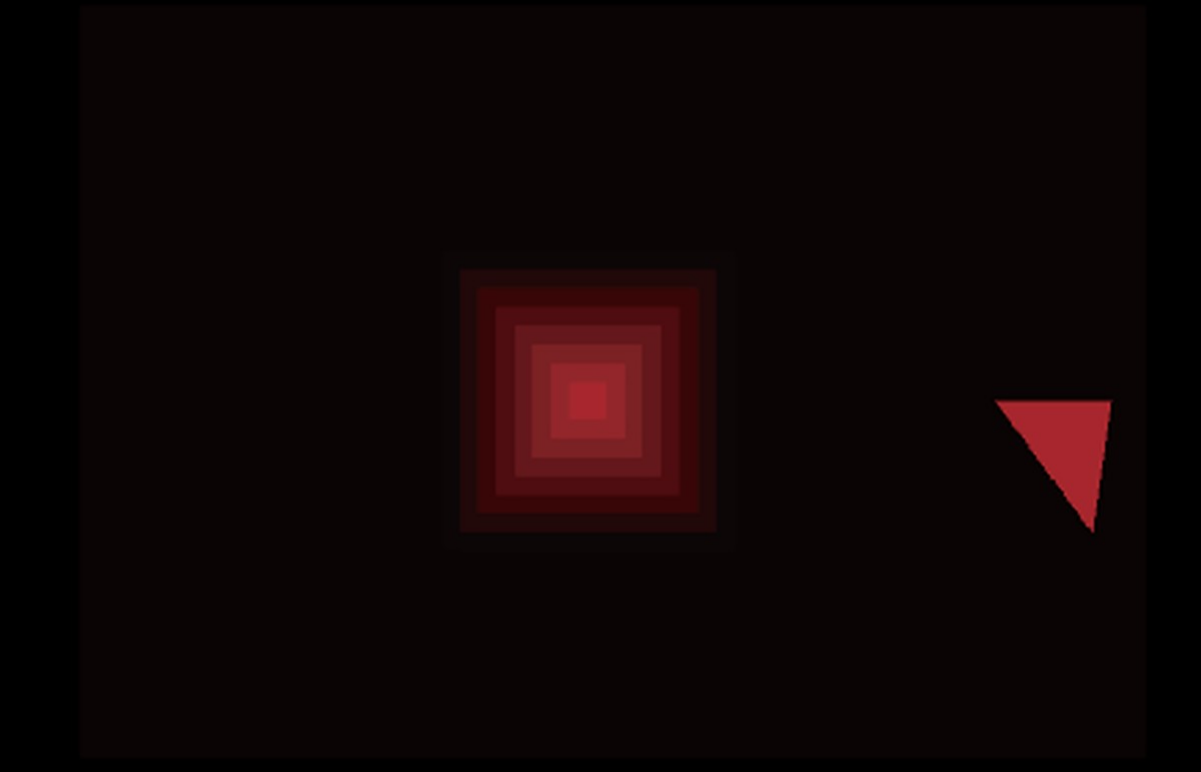
Stimulus in Experiment 1. The visual stimulus in Experiment 1. In the experiment it was viewed binocularly. The triangle beside the stimulus square is the arrow indicating the hand movement direction.

The visual stimulus was a four-sided pyramid, which was designed to be viewed from above (Fig 2). The pyramid was a flat square at the start of each trial, and the 3-D height of the pyramid changed in proportion to the distance that the stylus position moved. During this change, the apex of the pyramid was moved along the line of sight, which was perpendicular to the initial plane and passed through the center of the stimulus by adjusting to the participant’s head position (Fig 1). The pyramid was drawn as seven overlapping squares, and the edges of these squares provided binocular disparity to define the 3-D height of the pyramid. The 3-D height of the pyramid was defined only by binocular disparity, and its size did not change. The innermost square was used for fixation (to be precise, the pyramid was truncated, and its upper surface was the innermost square, but this innermost square was so small that it is referred to as the apex). The width of the largest square was 80 mm and was 6.5° at a viewing distance of 70 cm.

The haptic device was used for two purposes. One was to track the participants’ hand position, according to which the visual stimulus was updated. In each trial, the participants were required to move the stylus in one of two directions, that is, near (‘pull-toward’ condition) or far (‘push-away’ condition). During the movement, the 3-D height of the pyramid was updated in proportion to the distance of the stylus position from the initial flat plane. The participants were always required to push the stylus away or pull the stylus toward themselves along the line perpendicular to the flat plane from the initial position (i.e., the line accorded with the line of sight). The visual maximum 3-D height was determined in each trial, and it was one of the following values: ±1.2, 3.6, 6, 10, and 20 mm, where the positive values indicated that the surface protruded toward the observer. Each direction of hand movement (near/far) was paired with all visual depths, thus creating conflict conditions. Visual 3-D height was changed in proportion to the movement of the hand and attained maximum height when the distance of the stylus and the flat plane became 24 mm (e.g., when the maximum height was -10 mm, the 3-D height attained was -5 mm when the hand moved 12 mm in depth). The other purpose of using the haptic device was to generate force feedback to provide the participants with the impression of deforming rubber-like objects. The force, which simulated a spring force, had its anchor at the contact point, and its stiffness was 0.4 N/mm.

#### Procedure

The experimental trials were conducted in a dimly lit room. Participants could choose to either stand or sit in a height-adjustable chair. All participants were asked to put their forehead on a headrest placed on the upper side of the CRT (see Fig 1).

At the start of each trial, the participants were asked to grasp the stylus with their right hand (all participants were right-handed). After this, a visual stimulus appeared at the center of the display, and an arrow, which specified the direction toward which observers should move their hand, appeared on one side of the stimulus. The participants moved the cursor, which coincided with the point of the stylus, to touch the center of the square (fixation point). Once the cursor contacted the center of the square, the participants were informed of this by the haptic device, the participants moved their hand according to the direction indicated by the arrow. The haptic device restricted the movement in the opposite direction. The participants were required to move the stylus 24 mm in approximately 250 ms. The visual stimulus, which was defined by binocular disparity, protruded or became concave simultaneously with the stylus movement. Additionally, the lightness of the stimulus color synchronously increased by approximately 10% with stylus movement. This treatment was added because, without this treatment, participants could not be sure whether or not the stimulus was truly changing in conditions with small changes in visual height. This color change itself contained no information about the visual 3-D height.

The whole visual stimulus disappeared when the moved distance of the hand from the initial plane attained 24 mm. Then, the participants made a forced-choice response indicating whether the surface “visually protruded” or “was visually concave” by pressing the “6” or “4” key, respectively, on a 10-digit keyboard. Participants were instructed to respond based on their visual perception. No feedback on their responses was provided. Trials that took less than 150 ms or longer than 400 ms to move the stylus 24 mm were discarded and added to the sequence again.

In each session of Experiment 1, all combinations of two hand movement directions (push-away, pull-toward) and ten visual maximum 3-D heights were presented twice. Each participant performed four sessions, meaning that participants conducted all combinations of conditions eight times, except one participant who conducted five sessions. The order of the conditions (combining hand movement direction and visual 3-D heights) was randomized within each session and for each of the participants.

Before the actual experimental sessions, the participants performed practice trials. During the practice session, the participants learned the procedure, the appropriate moving speed of the stylus, and the appropriate direction of hand movement. After confirming that a participant could stably execute the trials, the experimental session was started.

#### Ethics statement

All experimental procedures in the present study were approved by the Ethics Committee for Human and Animal Research of the National Institute of Advanced Industrial Science and Technology (AIST) and given the approval number 2015–253. Written informed consent was obtained from all participants before the experiment.

#### Participants

Eight participants, five men and three women, aged between 20−39 years, with normal or corrected-to-normal vision, participated. Except for one participant, none of them knew the aim of the present experiment. All participants were right-handed.

#### Results

**Fig 3:**
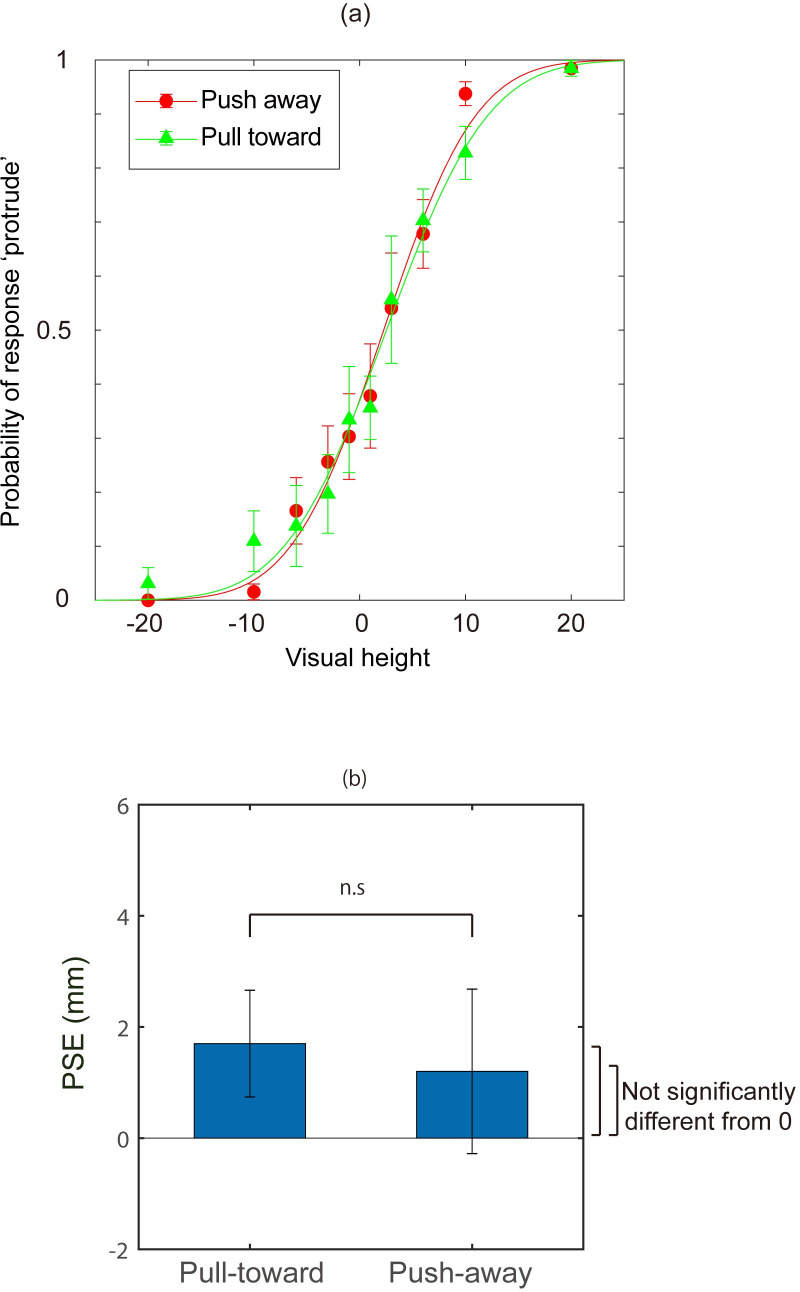
(a) The mean response probability perceived as “protrude” in Experiment 1. Error bars indicate mean standard error. Cumulative Gaussian functions fitted to both means are displayed. Green triangles indicate probabilities of perceived protrusion when the participants moved their hand for the “pull-toward” condition. Red squares correspond to the movement for the “push-away” condition. (b) The mean point of subjective equality (PSE) for all participants obtained from the results of Experiment 1. Error bars indicate the mean standard errors of PSEs among participants.

The probability of perceived protrusion was calculated from participants’ responses. Fig 3 shows the mean of the probability among all participants (Fig 3A). Moreover, to quantitatively evaluate the effect of the haptic information, a psychometric function was fit to an individual’s data to estimate the point of subjective equality (PSE) for each participant. To obtain psychometric functions, the data were fitted with a cumulative Gaussian function by maximum likelihood estimation methods. The averaged probabilities of stimuli judged as “protrude” across all participants are shown in Fig 3B. The mean PSEs among observers for each of the hand movement directions were 1.6 mm (SD = 0.97) and 1.1 mm (SD = 1.5) for the push-away condition and the pull-toward condition, respectively. A t-test revealed no significant difference between the two directions of hand movement (t (7) = 0.318, p>0.5, r = 0.12). Moreover, neither PSE was significantly different from 0 (t (7) = 1.77, p = 0.12, for the push-away condition; t (7) = 0.977, p = 0.36, for the pull-toward condition). The just noticeable difference (JND; difference between p = 0.75 and p = 0.5) was also calculated from the data, and the JNDs were -4 (SD = 2.12) in the push-away condition and -5.1 (SD = 3.47) in the pull-toward condition. A t-test revealed no significant difference between the JNDs for the two directions of hand movement (t (7) = 1.002, p = 0.35, r = 0.35).

**Fig 4:**
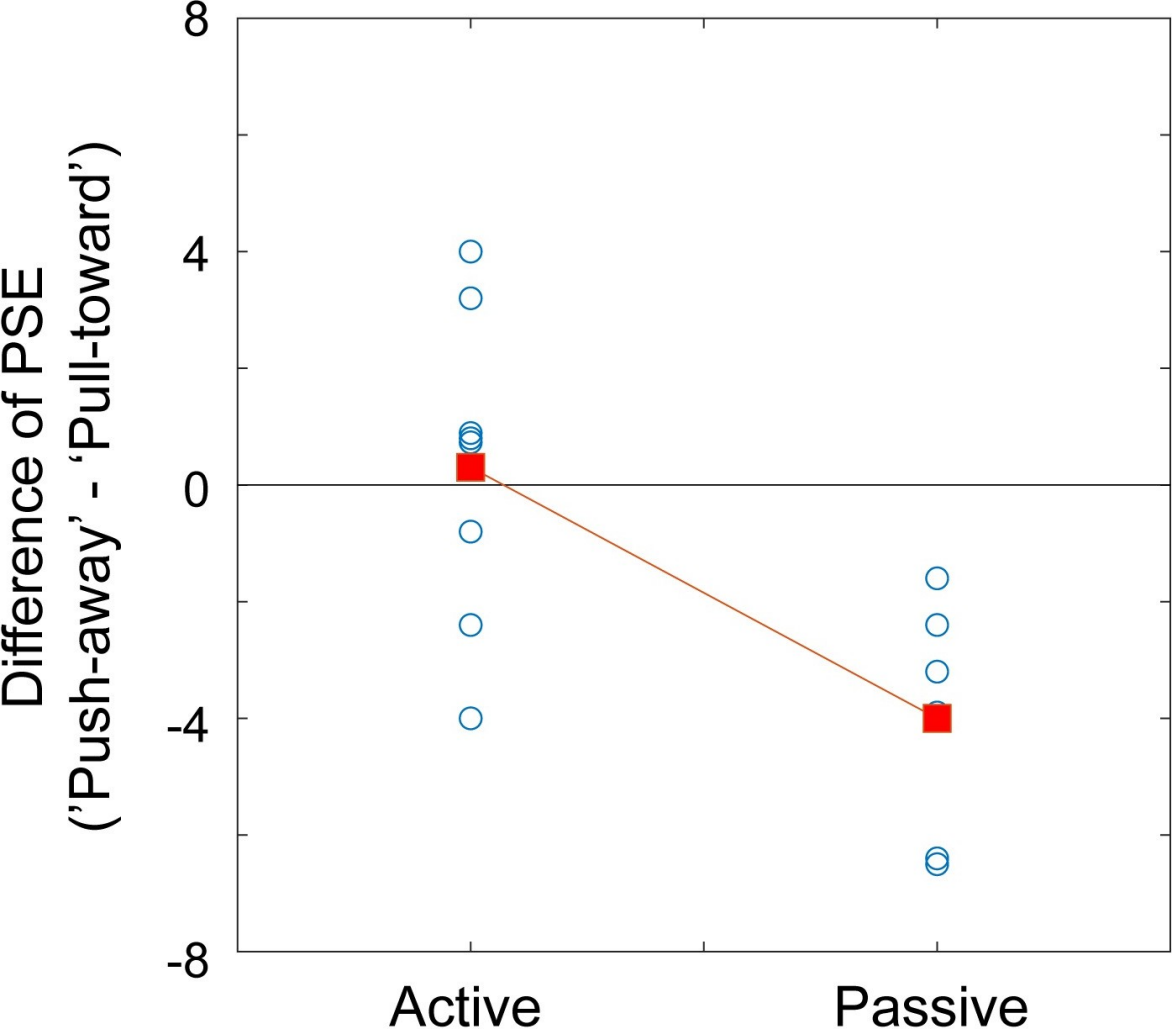
Comparison of the difference in point of subjective equality (PSE) between two movement directions in the present experiment (active condition) and in a previous study (passive condition) [15]. The blue circles indicate the differences for individual participants (some of them overlap). The red squares indicate the mean of the PSE difference in each of the two conditions.

The results showed that active hand movement did not affect visual depth perception. This is inconsistent with an earlier study [15], which examined the effect of passive hand movement on visual perception; here, perceived 3-D height was biased toward the direction of hand movement. Since the data from individual participants in this previous study [15] are available online, an additional analysis was conducted to compare the effects of hand movements by calculating the differences between the two directions of movement in the present study and the previous study. Fig 4 shows these differences (PSE in the push-away condition minus PSE in the pull-toward condition) in the two studies. In [15], PSEs were 0.46 (SD = 1.33) for the pull-toward condition and 4.46 (SD = 2.65) for the push-away condition (in [15], the hand was moved toward observer in the ‘push-back’ condition and this condition corresponds to the pull-toward condition in the present study. While the ‘pull-away’ condition in [15] corresponds to the push-away condition). An unpaired t-test was conducted for these differences, and the results showed that there was a significant difference between the effects of hand movement in these two studies (t (13) = -3.572, p = 0.03, r = 0.7). JNDs were also compared using unpaired t-tests, in which the push-away direction and the pull-toward direction were separately compared. In [15], JNDs were -3.8 (SD = 2.33) for the push-away condition and -4.6 (SD = 2.65) for the pull-toward condition. The results showed that there was no significant difference in either condition (t (13) = -0.139, p>0.5, r = 0.04; t (13) = -0.272, p>0.5, r = 0.08, respectively). Since the samples in both studies were small and unpaired, it seems that more data are required to confirm the absence of a difference in JNDs. This analysis with the two small samples, however, revealed that the effects of hand movement on PSE differed between active and passive movement conditions.

#### Discussion

The results of Experiment 1 showed that active hand movement did not affect perceived depth direction, which is different from the results obtained in a previous study with passive hand movement. In both studies, the trajectories and duration of the stylus point movement were nearly the same, and the relationship between hand movement and depth change in the visual stimulus were the same. Only difference between two studies was that the participants were not informed in advance of the direction of the hand movement in each trial in the previous study.

Here, the factor which I confidently consider as a source of difference in the results is that active hand movement could produce more precise proprioceptive information because an efference copy would also be available for the brain [16]. In the present experiment, the brain compared the distance of the hand movement with the perceived visual depth change. If a discrepancy between them was detected, then information from the two modalities would not be combined. Whereas, when hand movement was passive [15], the haptic signal itself was less accurate. In such a case, the brain might not separate these signals from different sources because it would be difficult to confidently detect a discrepancy. As a result, in that case, information from the hand movement affected visually perceived depth.

If the separation of signals was promoted by precise proprioceptive information, then it implies that if the visually presented depth information does not conflict with the distance of hand movement, an active hand movement should bias visual depth perception toward the depth indicated by the hand movement. To examine this, a different experimental paradigm involving disambiguation of depth direction (concave or convex) provided by a shading cue was used in Experiment 2. It is known that ambiguity in shape-from-shading occurs when a Lambertian surface is illuminated under collimated lighting and viewed under orthographic projection. Under such conditions, the same shading pattern results from the surface that has reversed depth and is illuminated from the opposite side [17]. The ambiguity is thought to be resolved by the brain based on prior assumptions such as assuming that the illumination comes from above (light-from-above prior [18–20]) or that objects are globally convex (convex-prior [17,21]).

By employing stimuli defined by shape-from-shading, it is possible to render stimuli whose depth does not conflict with hand movement and to examine the effect of hand movement by testing its perceived depth direction. If hand movement is effective for depth perception, the perceived depth direction would shift toward the direction of the hand movement. Moreover, by using a shading cue, it is expected that it would be more difficult to detect a discrepancy between the hand movement and visually given depth information because the perceived (absolute) depth obtained from the shading cue is unreliable [21]. This unreliability was strengthened in Experiment 2 by adding noise to the surface, since it was expected that it would be suitable to assess the effect of active hand movement in this situation.

## Experiment 2

In Experiment 2, the shape of the surface was defined by a shading cue. As in Experiment 1, participants pushed a stylus away or pulled a stylus toward themselves on a surface, and the visual stimulus simultaneously changed. The direction of the light was controlled as a variable to observe the relationship between the contributions of hand movement and other priors to the perception of visual depth.

## Materials and method

### Apparatus and stimuli

**Fig 5:**
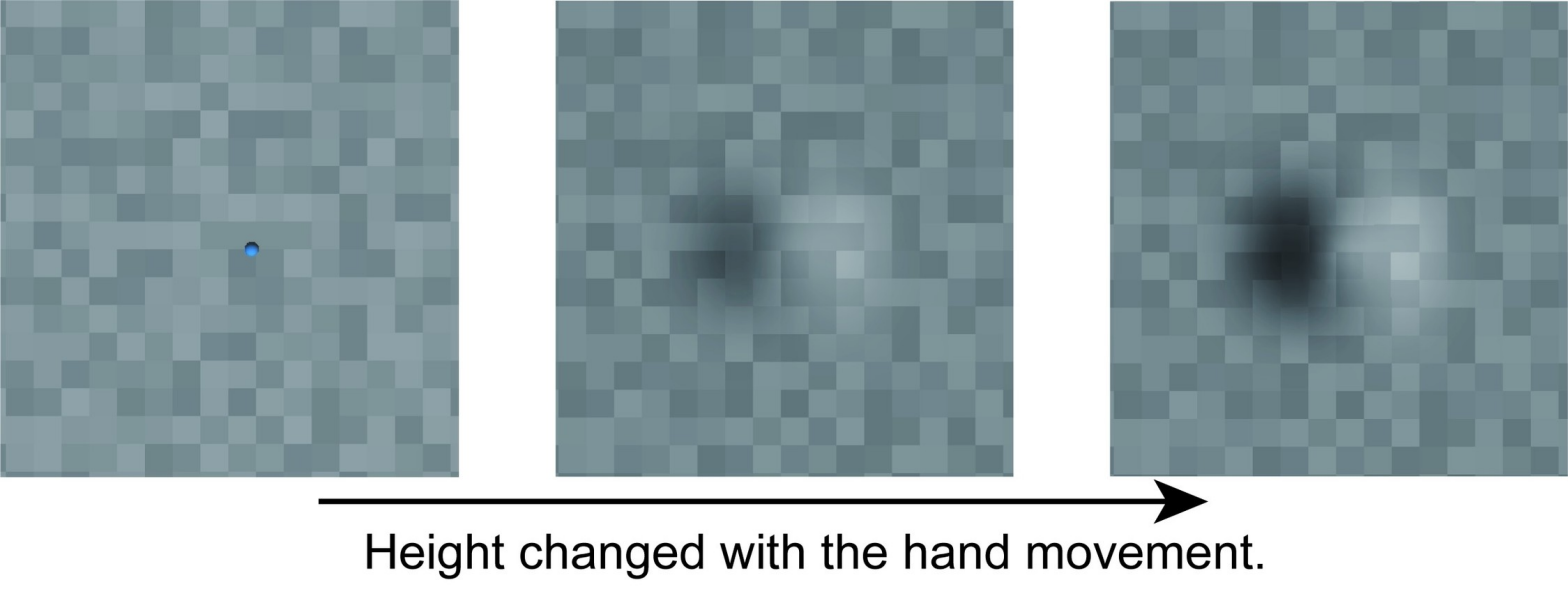
Stimuli in Experiment 2. The 3-D height of a curved surface drawn with shading cues changed with participants’ hand movements. In this figure, light source direction is 90° with the pull-toward hand movement as well as 270° with the push-away hand movement (The results with 270° were converted into 90° in the analysis). The blue dot in the leftmost image is the cursor that indicates the position of the point of the stylus. It disappeared during stimulus change.

The stimulus was a shaded curved surface on a surface covered with patches of random luminance, as depicted in Fig 5. The curved surface was generated using a nonuniform rational B-spline (NURB), and its shape was similar to that of a 3-D Gaussian. Both its width and height were approximately 6 cm (5° in visual angle), and its 3-D height was smoothly changed from 0 to 24 mm (note that the 3-D height here is a parameter for rendering shading gradient) with observers’ hand movement. The surfaces were grayscale random colored squares, and the reflectance of each patch was randomly chosen from 0.5 to 1, where 0 is black and 1 is white. The surface had no specular components. The light source direction was chosen from 0° (from above) to 360° in 30° increments. Convex surfaces made by “pull-toward” hand movements generated the same images as concave surfaces made by “push-away” movements lit by an opposite light source.

The experimental setting and apparatus were the same as in Experiment 1. Although the stimuli inherently contained a monocular depth cue, participants also wore stereo shutter glasses in Experiment 2 because they needed to place the cursor on the surface in 3-D space before performing the “push-away” or “pull-toward” movements on the stylus to change the visual stimulus. The participants touched a point around the center of the surface, and then a visual change was produced at the touched position. This change resembled deforming a rubber surface using the stylus (if the direction of visual 3-D height change and hand movement agreed). During the 3-D height change, visual stimuli were monocularly provided by closing the liquid shutter on the left eye of the stereo shutter glasses. Since the surface was laid on a plane of zero disparity, the participants would not notice any incongruity when the shutter was closed.

The procedure in Experiment 2 was as follows. The participants touched a flat surface and moved their hand according to the designated direction by a triangle slanted in depth appearing on the side of the surface. Then, the 3-D height of the curved surface changed with hand movement. The visual stimulus disappeared when the distance of the hand movement exceeded 24 mm or 800 ms following the onset of a change. After the visual stimuli disappeared, the participants answered whether the surface appeared as a bump (convex) or as a dent (concave). Trials that took less than 150 ms or longer than 400 ms to move the stylus 24 mm were discarded and added to the sequence again. The sample sequences of trials are shown in the supporting information items, titled S1 and S2 Movies.

As a reference in Experiment 2, a visual-only condition was prepared, in which the 3-D height of the visual curved surface was automatically changed at the position of the stylus when the stylus touched the surface. The change lasted for 250 ms.

In the sessions with hand movement, all combinations of the two movement directions (push-away, pull-toward) and fifteen light directions were presented eight times. The trials with hand movements were divided into two sessions. The visual-only session was conducted once. The order of the stimuli (combination of movement directions and/or light directions) was randomized within each session for each participant. Before the sessions, the participants performed practice trials with at least 10 push-away and pull-toward trials. After confirming that a participant could stably execute the trials, the experimental session started.

#### Participants

Thirteen participants, eight men and five women, aged 20−39 years, with normal or corrected-to-normal vision, participated. Except for one participant, none of them knew the aim of the present experiment. All participants were right-handed.

#### Results

**Fig 6:**
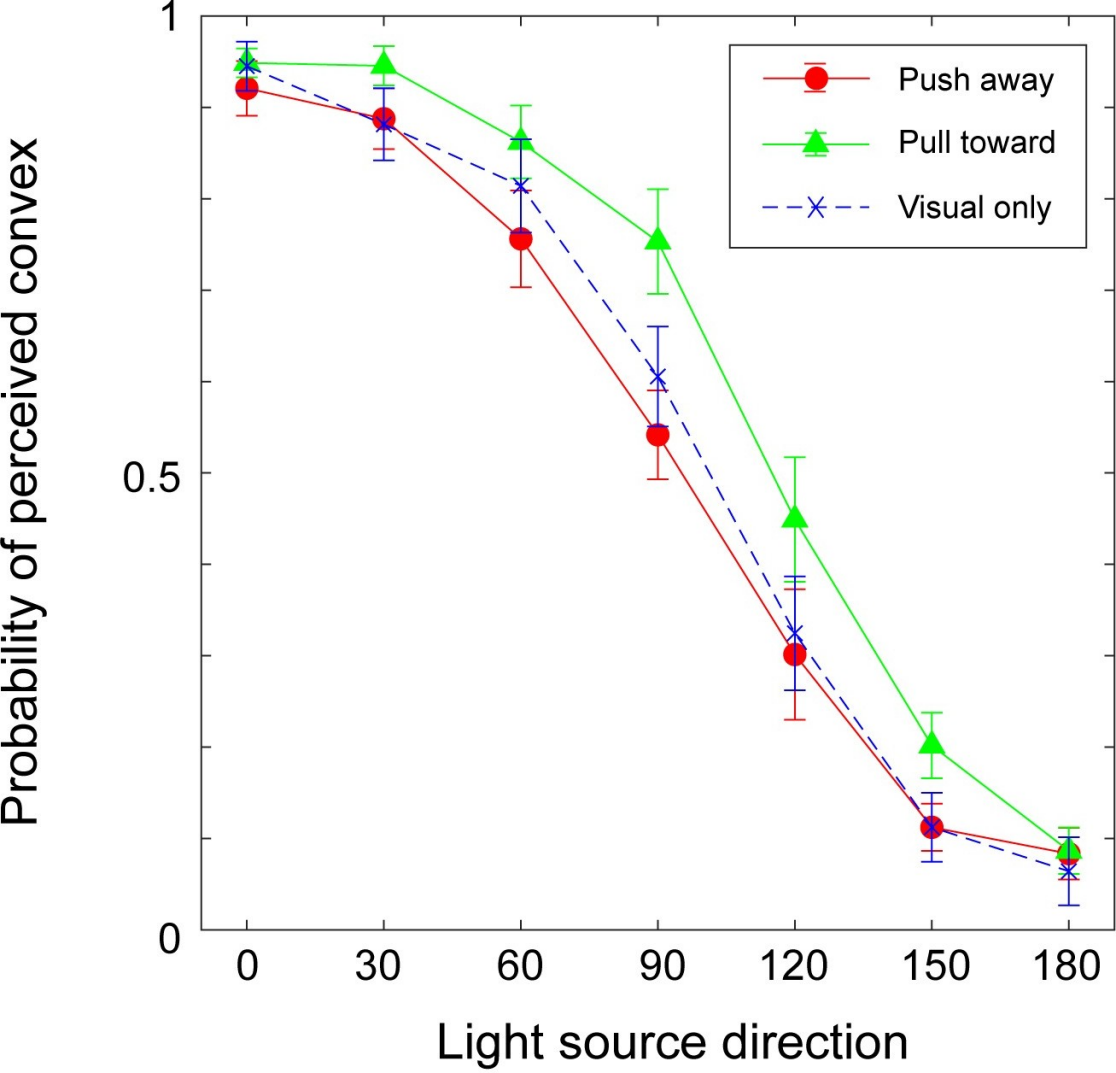
Results of Experiment 2. The probability of the surface being perceived as “convex” is plotted. The horizontal axis indicates the position of the light source in the visual stimuli assuming that the surface is convex.

In the analysis, responses were aligned according to the direction of illumination for the convex surface (i.e., pull-toward hand movement condition). This means that the responses for concave surfaces made by push-away movement were compared with the response to convex surfaces in the opposite direction. Moreover, the side of the illumination was not considered in the analysis. For example, responses to 120° in the push-away condition were combined with 240° and compared with responses for 60° in the pull-toward condition, which was the combination of the results of 60° and 300°. By doing so, it was possible to compare the two hand movement directions for the same shading pattern. Averaged probabilities of stimuli judged as “bump (convex)” among all participants are shown in Fig 6, obtained in two hand movement directions and the visual-only condition. Consistent with previous findings, the probability of the stimuli being perceived as convex generally decreased with a decrease in the direction of the light. Statistical analysis was applied to averaged probabilities with two-way ANOVA; the direction of hand movement (pull-toward or push-away) and the direction of light source (0° to 180°) were taken as the two factors.

ANOVA for the session with hand movement revealed a significant main effect of the direction of hand movement (F (1, 12) = 8.16, p = 0.01, η^2^ = 0.405), light source direction (F (6, 12) = 128.1, p<0.001, η^2^ = 0.914), and the interaction between the direction of hand movement and the light source direction (F (6, 72) = 2.82, p = 0.016, η^2^ = 0.190). Post hoc comparisons with Bonferroni correction were conducted on the effects of hand movement among different light directions. The results showed a significant effect of hand movement when the light directions were 60°, 90°, and 150° (p = 0.007, p = 0.008, and p = 0.044, respectively). Additionally, two-way ANOVAs were conducted to reveal the difference between each of the results concerning the direction of hand movement and visual-only condition. ANOVA between the results with pull-toward hand movements and those in the visual-only condition revealed a significant difference between these two conditions (F (1, 12) = 7.084, p = 0.02, η^2^ = 0.371) and the interaction between the direction of hand movement and the light direction (F (6, 72) = 1.051, p = 0.04, η^2^ = 0.08). Post hoc comparisons with Bonferroni correction revealed a difference when the light direction was 45° (p = 0.024). ANOVA between the results with push-away hand movements and those in the visual-only condition revealed no significant difference (F (1, 12) = 0.504, p = 0.49, η^2^ = 0.04).

#### Discussion

The results of Experiment 2 showed that hand movement affected the perceived direction of depth changes defined by the shading cue. The bias in these perceived directions was in accordance with the direction of hand movement. That is, the perceived depth direction was affected by active hand movement when the perceived visual depth provided by the shading cue did not conflict with the distance of hand movement.

The results also showed that the effect of hand movement on the perceived depth direction was weakened at approximately 0° and 180°. If the integration process obtained here is a kind of simple weighted summation, we would expect the effect of hand movement to exist across all light directions. This indicated that hand movement information was used as a disambiguation cue in this experiment. That is, the effect of hand movement might emerge when the light-from-above prior is not available to resolve the ambiguity.

Concerning disambiguation, previous studies reported that ambiguous depth direction defined by structure from motion (SFM) or texture cues was disambiguated by cues that conveyed depth direction or depth order information, for example, binocular cues and occlusion [22,23]. Here, we should note that the effect of hand movement on the perception of depth direction was relatively weak compared to the effects of binocular cues on the texture or that of occlusion cues on the SFM. This is likely because, unlike in the within-modality situation, the brain can easily interpret that the two relevant events coming from different modalities do not share the same source even though the hand movement and the visual depth change are coincident. When both cues are visual, separating such cues results in the existence of a transparent or a complicated surface, and such interpretations would be less plausible. However, when the cues of interest arise from different modalities, they do not pose a problematic interpretation for the brain, especially when information is given under an artificial setting.

When compared with the visual-only condition, the effects of the hand movement were significant in the “pull-toward” direction in which an observer would perceive that a surface with high viscosity was associated with their hand movement, and then the surface depth would be more frequently perceived as convex. In the following section, I will discuss this asymmetry.

As discussed in the Introduction, determining whether two signals originated from the same source can be explained with a causal inference model based on the Bayesian framework. Thus, it is expected that the findings of this study can also be explained using this framework. When hand movement is generated actively and binocular disparity provides a reliable estimate of absolute distance in the visual display, both estimations are expected to be reliable, and any difference between them is also supposed to be reliable. In this case, the brain judges that information from different modalities must have different sources. In the case of passive hand movement, proprioceptive information was degraded, and therefore, these two estimates may be integrated. In the following section, I tested this account for the present study’s results with a statistical model based on the causal inference model for perceptual processes.

### Simulation

Until recently, various types of models have been proposed for causal inference in perception based on the Bayesian framework, and it has also been pointed out that they have certain similarities [14]. From among these models, the present simulation referred to the mixture model proposed in previous studies [24–26]. The mixture model can switch between different models of the prior constraint according to the conflicts among estimations from multiple cues, even though the structure is not hierarchical.

In the Bayesian approach, when the information *I* is provided to an observer about a property of world *S*, the posterior distribution *P(S|I)* is given as the product of a likelihood function *P(I|S)* and a prior probability distribution *P(S)*:

P(S|I)=P(I|S)P(S)/P(I).

For most practical purposes, we may consider *P(I)* to be a normalizing constant. In the general formulation of the multimodal integration, there are multiple values of *I* and *P(I|S)* as the joint probability *P(I*_*h*_,*I*_*v*_,*|S)*, where *I*_*h*_ represents haptic information and *I*_*v*_ represents visual information. *P(I*_*h*_,*I*_*v*_,*|S)* is decomposed into *P(I*_*v*_*|S) and P(I*_*h*_*|S)*, where the former is the visual likelihood and the latter is the haptic likelihood. The mixture model represents *P(I*_*h*_*|S)* by an additive mixture of the density function, which corresponds to either a causal situation or a noncausal situation.

$$P\left(S|I_{v},\;I_{h}\right)=P\left(I_{v}|S\right)P\left(I_{h}|S\right)P(S)=P\left(I_{v}|S\right)\left[P\left(I_{h}|S,C_{1}\right)+P\left(I_{h}|S,C_{2}\right)\right]P\left(S\right)$$ (1)

Here, *C*_*1*_ corresponds to a situation with a single cause, and *C*_*2*_ corresponds to a situation with different causes. In this model, *P(I*_*h*_*|S*,*C*_*1*_*)* in Eq 1 is given by a Gaussian distribution around the hand movement distance. *P(I*_*h*_*|S*,*C*_*2*_*)* is given by a uniform distribution that represents a noncausal situation; therefore, haptic information has no effect on the visual estimation.

### Simulation of Experiment 1 (Simulation 1)

With this model, I examined whether the difference between passive and active movement observed in Experiment 1 could be simulated. We have three parameters to estimate in the simulation. One was the standard deviation of the Gaussian distribution, σ_*h*_, for *P(I*_*h*_*|S*,*C*_*1*_*)*, which corresponds to the precision of the haptic signals. We can expect that the haptic signals should be more precise in the active condition; that is, σ_*h*_ would be smaller in the condition. The mean of the distribution was the distance of the hand movement. The second was the width of the tail (uniform distribution), ω, for *P(I*_*h*_*|S*,*C*_*2*_*)*. This width of the tail ω controls the ease of separation of haptic and visual signals. In the present simulation, a common ω was given to both the active and passive conditions. The remaining parameter is the standard deviation σ_*v*_ of the Gaussian distribution for *P(I*_*v*_*|S)*. The mean of this distribution was the value of the given visual 3-D height.

**Fig 7:**
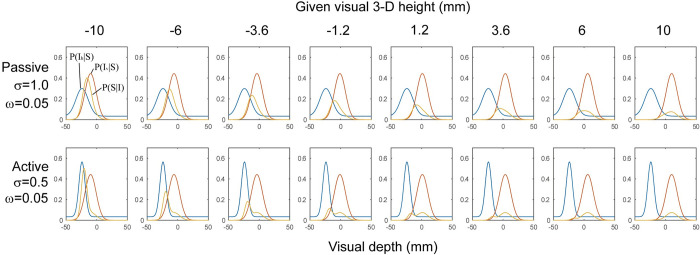
Likelihood distributions in Simulation 1. Plots in the upper row show distributions used in the simulation in the passive conditions with selected given visual 3-D heights. Plots in the lower row show distributions in the active conditions. In each plot, blue, red, and yellow curves correspond to *P(I*_*h*_*|S)*, *P(I*_*v*_*|S)* and *P(S|I)*, respectively. The y-axis of each plot is probability used in the simulation. Because the distributions are normalized, the width of the tail in these plots are different from ω displayed in the left.

Fig 7 shows the shapes of these likelihood functions (calculated with parameter values that are estimated later) and the resulting *P(S|I*_*v*_,*I*_*h*_*)*. With resulted distributions of *P(S|I*_*v*_,*I*_*h*_*)*, we can calculate ratios of areas above 3-D height>0 (corresponding to the response “protrude”) and 3-D height<0 (corresponding to the response “concave”) for each of the visual 3-D heights, and then these can be compared with the results obtained in Experiment 1.

The mean squared error between the model estimation and the response probability of each participant in Experiment 1 was calculated to fit the parameters of the model. The range of interest of depth was set from -50 mm to 50 mm, and each distribution was normalized to be its area become 1 within the range. The mean squared errors were calculated at ten visual 3-D heights that were the same as those used in Experiment 1. Parameters were tested among ranges of σ_*h*_ = {0.1… 2.0} at 0.1 increments, ω = {0…0.9} at 0.025 increments, and σ_*v*_ = {0.1….1.5} at 0.1 increments. In this model fitting, a combination of σ_*v*_ and ω that resulted in the lowest sum of mean squared errors in the active and passive conditions was searched for first. This procedure was chosen because σ_*v*_ and ω were assumed to be the same between the active and the passive conditions in the simulation. With these σ_*v*_ and ω, σ_*h*_ for the active and passive conditions were found.

**Fig 8:**
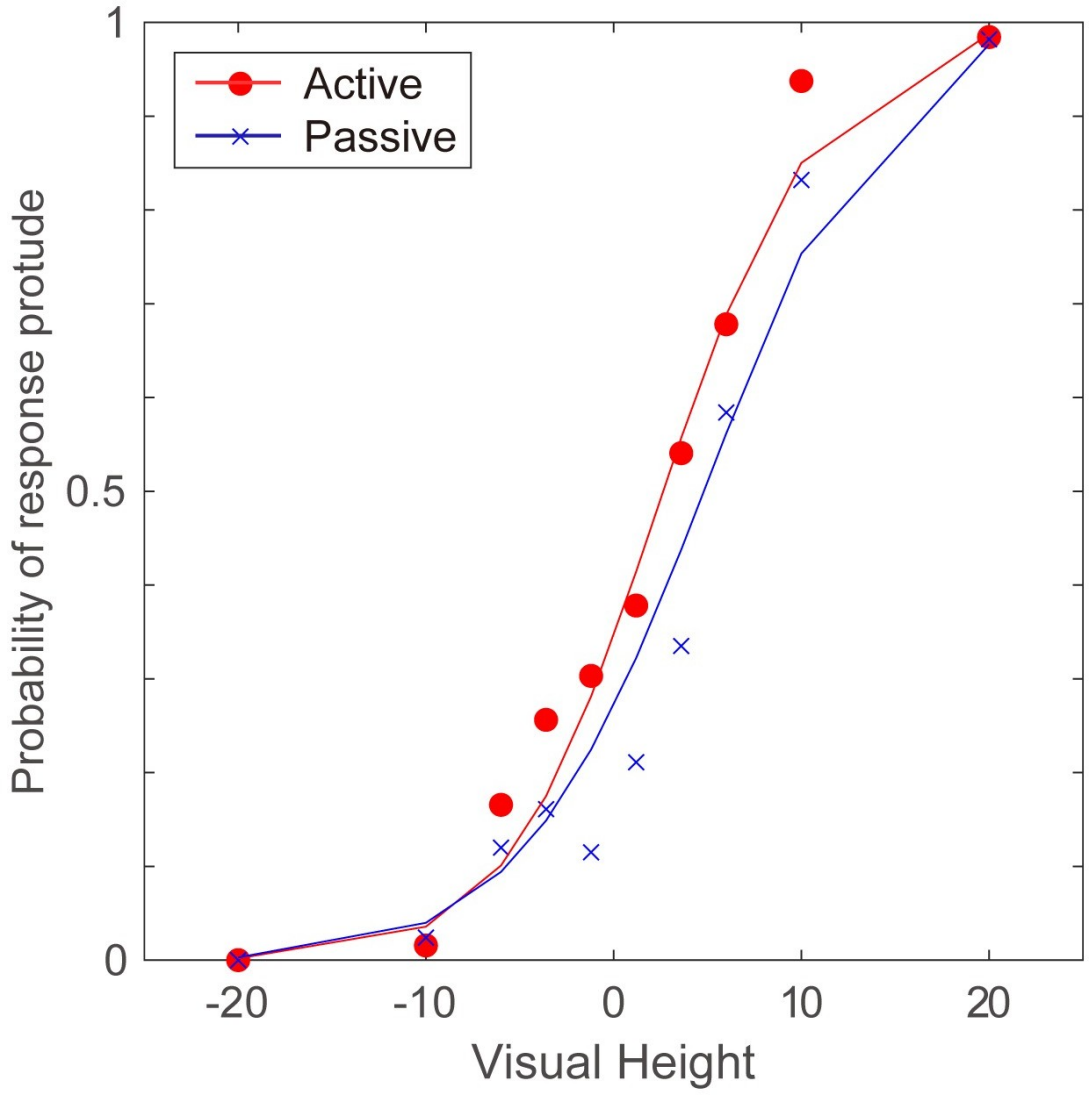
Results of Simulation 1. Estimated probability of being perceived as protruding against the given visual 3-D height in Simulation 1. The red line corresponds to the active condition, and the blue line corresponds to the passive condition. Triangles and crosses indicate mean response probabilities from Experiment 1 and [15]. Only the results for the push-away condition are displayed here.

As a result, I obtained the following set of parameters: σ_*h*_ = 0.5 for the active condition, σ_*h*_ = 1.0 for the passive condition, ω = 0.05 and σ_*v*_ = 0.9. The estimated response probabilities are shown in Fig 8 with the mean response probabilities from Experiment 1 and [15], which shows that the model was fitted well. This result indicates that the model could replicate two aspects of the experimental results. One is that in the passive setting, the observed 3-D height was biased toward the direction of hand movement. The other is that the active hand movement had nearly no effect on perceived 3-D height. As expected, a smaller σ_*h*_ was estimated for the active condition, which reflects the greater precision in the active hand movement.

### Simulation of Experiment 2 (Simulation 2)

Next, Experiment 2 was simulated using the same framework. The structure of the model for Experiment 2 was the same as in Eq 1. The hand movement generated in Experiment 2 was also represented by the mixture distribution of Gaussian distribution with the standard deviation of σ_*h*_ and the uniform distribution with the width of ω was given to *P(I*_*h*_*|S)*.

However, the distribution for *P(I*_*v*_*|S*,*C)* differed in Experiment 2. *P(I*_*v*_*|S*,*C)* for Experiment 2 was given by the summation of two Gaussian functions whose means were the same as the distance of hand movement (+/-24 mm). This reflected the ambiguity of perceiving depth direction in shape from shading. Hereafter, these distributions are referred to as *p*_concave_ and *p*_convex_.

**Fig 9:**
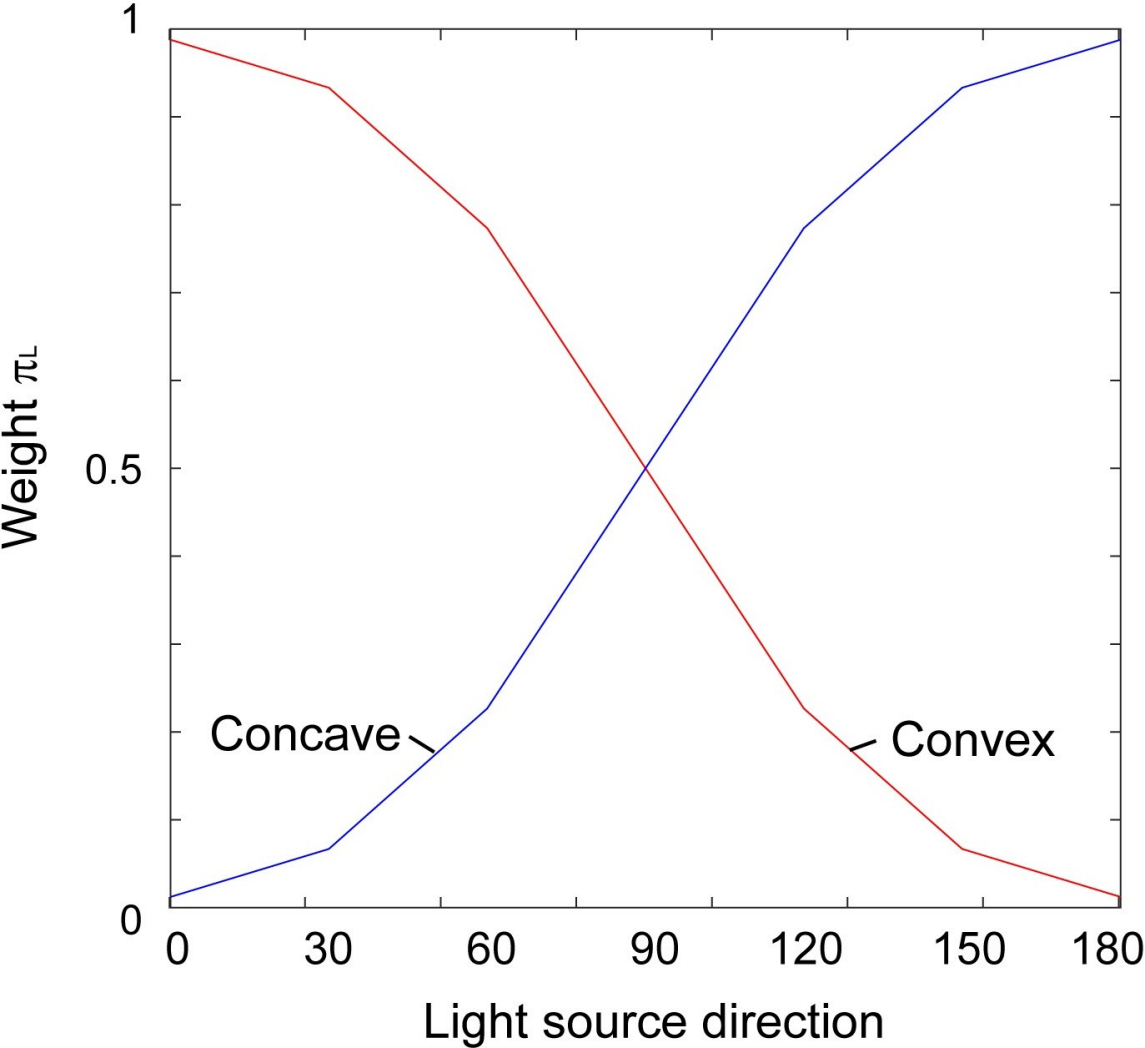
Weights π_L_ used in the simulation. The π_L_ for the distribution around -24 mm (*p*_concave_) is shown as a red line and shown as a blue line for the distribution around 24 mm (*p*_convex_).

In addition to this, the contribution of the prior concerning shape from shading were incorporated by assigning a weight, π, which modulated the relative height of *p*_concave_ and *p*_convex_. π was comprised of two priors; regarding the light source direction (i.e., light comes from above) and regarding a surface convexity [17,21]. The component regarding the light direction (π_L_) were given by distributions shown in Fig 9. π_L_ for *p*_concave_ is shown as a blue line in Fig 9, and for *p*_convex_ is shown as a red line. A cumulative Gaussian distribution was used to represent the distribution, and the distributions in Fig 9 are drawn with the standard deviation estimated below. In this figure, the horizontal axis indicates the direction of illumination with which direction and a convex surface generates an image (i.e. the same as the horizontal axis in Fig 6). Weights represented by each line in Fig 9 correspond to the preference to interpret the generated image with the light source direction perceived as concave or convex.

The component regarding the preference to perceive convex surfaces, which was observed in the response for the light from the side (90°) in the visual-only condition, was also incorporated (π_C_). The π_C_ was constant regardless of the change of the light direction.

**Fig 10:**
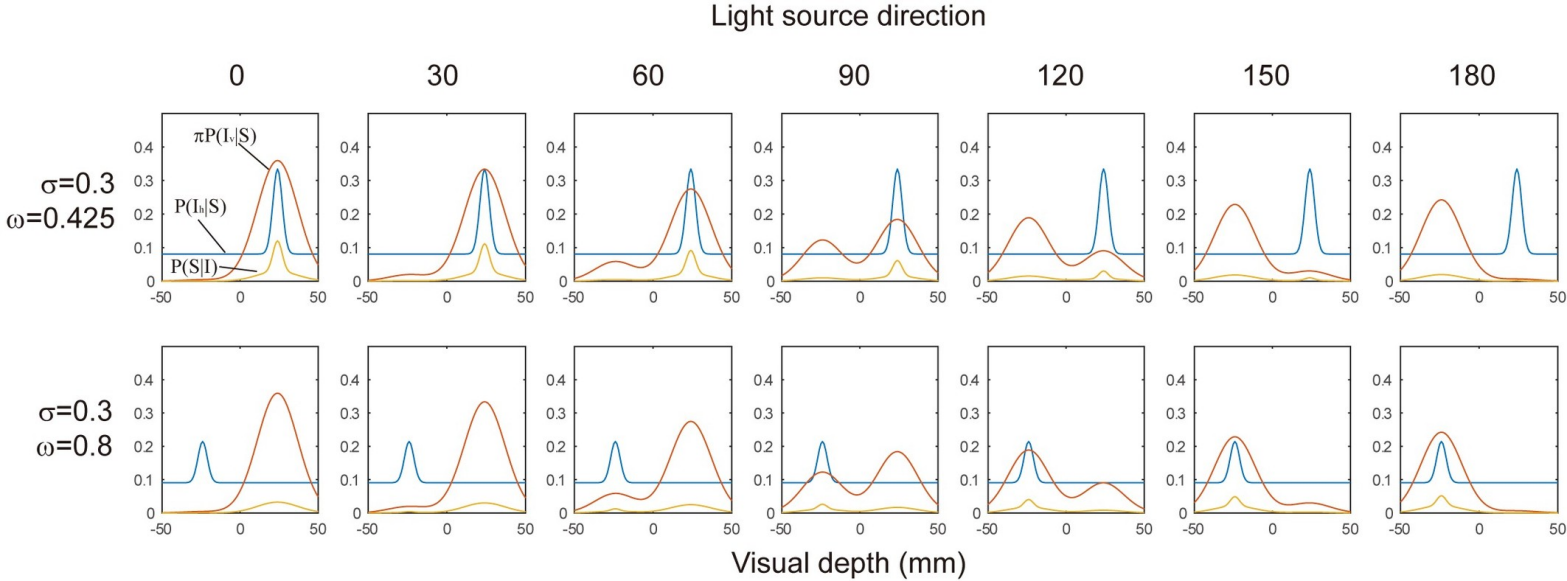
Likelihood distributions in Simulation 2. Distributions used in the simulation for each of the light source positions in the visual stimuli assuming that the surface is convex. Plots in the upper row show distributions used in the simulation for pull-toward conditions. Plots in the lower row show distributions for the push-away condition in which the effect of the hand movement was weak. In each plot, blue, red, and yellow curves correspond to *P(I*_*h*_*|S)*, π*P(I*_*v*_*|S)*, and *P(S|I)*, respectively. The y-axis of each plot is probability used in the simulation. The distributions are normalized, therefore the width of the tail in these plots are different from ω displayed in the left.

In the simulation, π was given as a product of π_L_ and π_C_. π was calculated for *p*_concave_ and *p*_convex_ with a given light source direction, and multiplied to each corresponding distribution. Then, *p*_concave_ and *p*_convex_ were summated. Here, the standard deviations for *p*_concave_ and *p*_convex_, were the same (σ_*v*_). Fig 10 shows the shapes of these likelihood functions and the resulting *P(S|I*_*v*_,*I*_*h*_*)*. The ratios of areas above 3-D height>0 (corresponding to the response “protrude”) and 3-D height<0 for each of the visual 3-D heights were calculated and then they were compared with the results of Experiment 2.

As conducted in Simulation 1, the mean squared errors between the model estimation and the response probability of each participant in Experiment 2 were calculated to find the parameters to fit the model. We first determined three parameters to fit data in the visual-only condition. These parameters were π_C_, σ_*v*_, and the standard deviation for the cumulative Gaussian function for π_L_. The simulation was conducted for seven light directions, which were the same as those in Experiment 2. This fitting gave 1.2 for π_C_ for *p*_convex_, 0.8 for *p*_concave_, 1.3 for σ_*v*_ and 0.7 for the standard deviation of cumulative Gaussian functions. Then, σ_*h*_ and ω were estimated against the results of the pull-toward conditions.

**Fig 11:**
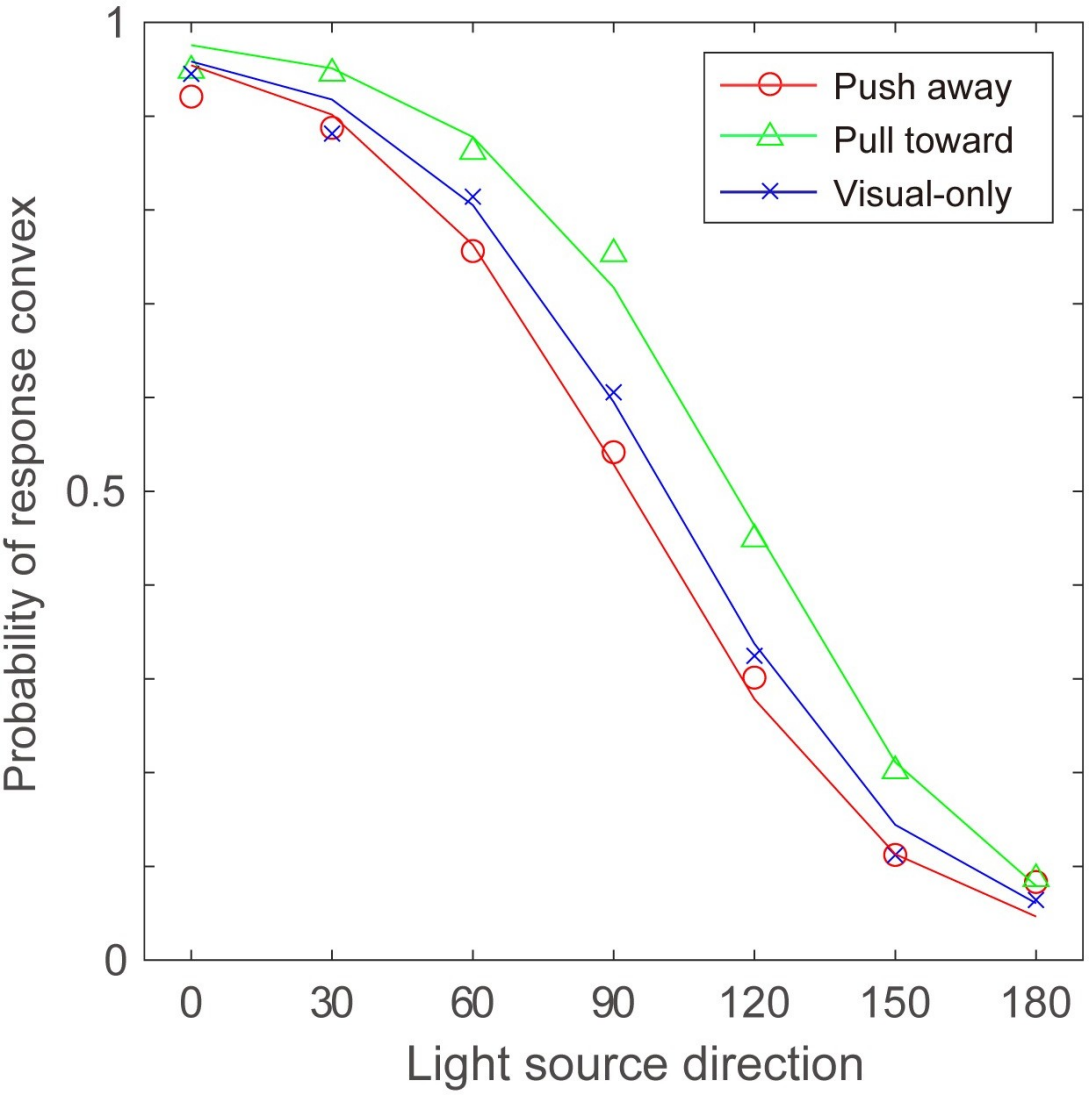
Results of Simulation 2. Estimated probability of being perceived as convex against the light direction in Simulation 2. The color of each line corresponds to the results of the pull-toward condition (σ = 0.3, ω = 0.425, green) and push-away (σ = 0.3, ω = 0.8, red) and visual-only (blue) conditions. Triangles, circles and crosses indicate mean response probabilities from Experiment 2.

The green line in Fig 11 shows the results of the simulation for the pull-toward condition with the best fitted parameters; σ_*h*_ = 0.3 and ω = 0.425. As in Simulation 1, each distribution was normalized, therefore ω did not directly correspond to the numerical probability in the distributions. These simulation results demonstrated that the perceived depth directions were biased toward the hand movement directions. The effect of hand movement could be replicated with the likelihood distribution in which visually ambiguous and unreliable situations were represented.

The results of Experiment 2, however, showed an asymmetrical effect of hand movement. Although the experimental results did not show a significant difference, the results could be replicated with the increase in the parameter σ_*h*,_ which is shown in the lower part of Fig 10, and the results are displayed in Fig 11 with a red line. By increasing σ_*h*_, the distribution approaches flat, and as a result, the estimation approaches to the simulation results of the visual-only condition. It is hard to believe, however, that the reliability for the hand movement decreased so much when the hand movement direction was push-away. Another manipulation to replicate the asymmetry is increasing ω, which also flatten the distribution. To perceive a dent surface more frequently, visual changes other than shading gradients, such as shadows, might be expected by the brain, and then haptic information might be ineffective. The simulation with greater ω which implement the increased ease of separating two signals from different modality might represent such a case We, however, need further investigations to reveal additional aspects of the asymmetry and to examine the model description explaining this situation.

Lastly, I examined the necessity of the tail in this model. The tail was necessary for Simulation 1 because the model without the tail biased estimated depth toward the direction indicated by the hand movement when σ_*h*_ was small. This is different from the presupposition that haptic signals were more reliable when they were actively generated. While in Simulation 2, we need to confirm whether the addition of the tail was necessary to show that these two experiments could be explained in the same framework. We examined this by comparing the results of Simulation 2 for the pull-toward condition with σ_*h*_ = 0.3 and ω = 0.425 and the results of the estimation with ω = 0. The parameter value of σ_*h*_ for the latter was determined among {0.0 …. 2.0} at 0.1 increments (we restricted the range of σ_*h*_ because too large σ_*h*_ would spoil the meaning of the model), and 2.0 gave the most fit results. The least mean squared error for the model with the tail was 2.1 and that without the tail was 7.1. Because the number of parameters was different, a comparison with the Akaike information criteria (AIC) was conducted. This showed that the results were well fitted by the model with the tail (AIC = -161.5 for the model with tail and 12.5 for the model without tail). These results indicated that the causal inference model was well fitted to the experimental results obtained in Experiment 2.

### Summary of simulation

The results of the simulations showed that the experimental results could be accounted for using a mixture model with a Bayesian framework. The important notion in this simulation is that precise haptic information by controlling σ_*h*_ could replicate the degree of separation of the two signals in Experiment 1, and the same framework could replicate the disambiguation of depth from shading.

In the simulation of Experiment 2, the process corresponding to disambiguation was not apparently incorporated; however, the results showed good replication. Adams and Mamassian [25] showed that experimental results in which binocular cue disambiguated depth direction perception from monocular texture cues were replicated by a simulation with a Bayesian model that did not have an explicit cue promotion stage but incorporated prior information for convexity and flatness. I believe the present results are in line with this study. The disambiguation process could be accounted for by the process in which missing or tentative parameters are overwritten by information from other cue(s).

Although the simulation showed good fit to the experimental data, there remains several points we should investigate. One of them is that the width of the tail was largely different between two simulations. Though it might not be odd because the likelihood function for *P(I*_*v*_*|S*,*C)* was different, the same or similar haptic likelihood would be expected for the similar hand movement. In further studies, understating the meaning of the tail across various situations should be necessary. Another point we should investigate is understanding an asymmetric effect of hand movement directions. Although, the asymmetry in Experiment 2 was discussed above, the experimental results in [15] also showed an asymmetric effect of hand movement. It should be necessary to investigate the asymmetry from both the haptic and visual aspects in further studies.

## Summary

The first finding in the present study was that active hand movements had no effects on visual depth perception when combined with binocular depth cues. These results differed from those of a previous study, which showed that information from passive hand movement affected perceived depth [15]. Experiment 2 revealed that active hand movement affected the perception of depth direction when combined with visual depth information provided by a shading cue. This second finding suggested that if there is no conflict between visual information and hand movement, information from active hand movements helps the perception of ambiguous depth direction. These processes were reproduced by a simple model, which was a mixture model based on a Bayesian framework.

Finally, it is necessary to mention an important topic that should be examined in future studies. In Experiment 2, we did not include a passive hand movement condition. This is because our interest lies in whether or not active hand movement is effective for visual perception. It is necessary, however, to obtain a stricter comparison of active and passive situations within each participant to confirm the validity of the model and the speculations that predicted that the effect of hand movement would be stronger in the passive condition in Experiment 2. Individual differences should also be examined in further investigations. Individual differences can exist in sensory cue integration and causal inference (e.g., [26]), and accounting for individual differences can demonstrate the validity of the model. For this purpose, elaborations and modifications of the experiment may be needed to obtain precise individual data.

## Supporting information

S1 Table Probability of perceived 'protrude' for each participant in Experiment 1. (XLSX)

S2 Table PSE and JND for each participant in Experiment 1. (XLSX)

S3 Table Probability of perceived 'convex' for each participant in Experiment 2. (XLSX)

S1 Movie Sample stimulus sequence in Experiment 2 (light direction = 180°). (ZIP)

S2 Movie Sample stimulus sequence in Experiment 2 (light direction = 90°). (ZIP)
